# Geographic inequality in funding by National Institutes of Health negatively impacts almost one-half of the states in the United States

**DOI:** 10.3389/fpubh.2024.1452494

**Published:** 2024-09-25

**Authors:** Elizabeth Caulder, Jiajia Zhang, Mitzi Nagarkatti, Prakash Nagarkatti

**Affiliations:** ^1^School of Medicine, University of South Carolina, Columbia, SC, United States; ^2^School of Public Health, University of South Carolina, Columbia, SC, United States

**Keywords:** NIH (National Institute of health), funding, healthcare, health policy, geographic disparity

## Abstract

**Introduction:**

The National Institutes of Health (NIH) is the primary federal agency in the United States (US) that supports biomedical research, training, and clinical trials. NIH funding creates patents and jobs and thus helps the regional and national economy grow. Therefore, NIH funding would be expected to flow equitably to all 50 US states. However, there is a significant geographic disparity in the level of NIH funding received by various states. To that end, in 1993, authorized by Congress, NIH initiated a funding program called the Institutional Development Award (IDeA) to support states, called IDeA states, which received low levels of NIH funding. However, whether this approach has helped reduce the geographic disparity in NIH funding is unclear.

**Methods:**

In the current study, we analyzed data on various NIH funding mechanisms awarded to 23 IDeA states vs. 27 non-IDeA states, as identified by NIH. We compared these data to the population size, federal taxes paid, and the number of PhDs and Post-doctoral Fellows(PDFs) trained in IDeA vs. non-IDeA states.

**Results:**

The non-IDeA states received 93.6% of the total NIH funding, whereas IDeA states received only 6.4%. On average, one Institutional Training Grant was received for every 24 PhDs trained in non-IDeA states, while IDeA states received one such grant for every 46 PhDs trained. The non-IDeA states comprised 84.3% of the US population, whereas IDeA states comprised 15.7%. Thus, on a *per capita* basis, non-IDeA states received $120 from NIH, whereas IDeA states received $45 per person. For every million dollars contributed by the non-IDeA states toward federal taxes, they received $7,903 in NIH funding, while the IDeA States received only $4,617. For FY 2022, the NIH funding created an economic activity of $90.6 Billion in non-IDeA states and only $6.3 billion in IDeA states. When total NIH funding to the states was analyzed for the years 1992, 2002, 2012, and 2022, IDeA states received 4.7% of the total NIH funding in 1992, which increased to 7.2% in 2002 but dropped to 6.8% in 2012 and 6.5% in 2022. This demonstrated that IDeA states’ share of NIH funding remained relatively unchanged for the past 20 years.

**Discussion:**

Eliminating the geographic disparity in NIH funding is crucial for achieving equitable health outcomes across the US, and for the IDeA states to successfully train future generations of physicians and scientists, as well as grow the regional economy. Although the NIH IDeA programs have helped enhance the research capacity in IDeA states, the funding currently constitutes less than 1% of the total NIH budget. Thus, it is critical to increase NIH funding to IDeA states to improve health outcomes for all Americans.

## Introduction

Federal funding for research across universities in the United States has been instrumental in promoting innovation and building the country’s economy (1, 2). It is well-known that major discoveries from academic research have transformed the innovation landscape, significantly impacting society. Some examples include touch screens and lithium batteries, different types of antibiotics to treat infections, plant genetics to create crops that are more resistant to drought and infections, insulin to treat diabetes, and search engines such as Google, and the like (3). Thus, it is expected that all 50 US states will receive equitable federal research funding so that each state can develop its workforce and grow its regional economy. Unfortunately, this has not been the case. There are approximately 6 states in the United States that receive one-half of the Federal Research funding (4, 5) while there are over 25 states that together receive ~10% of the Federal funding.

This geographic inequality in research funding has been a concern for the US Congress and has stirred significant debate (6). When the National Science Foundation (NSF) was created, Congress asked the NSF to develop approaches “to strengthen research and education in science and engineering throughout the United States and to avoid undue concentration of such research and education (7).” To that end, the NSF initiated a program in 1978 called the Experimental Program to Stimulate Competitive Research (EPSCoR). The goal of this program was to help approximately 25 states, territories, and a commonwealth group that secured less NSF research funding with resources to build their research capacity to become more competitive. This program allocates ~$250 million/year to fund the EPSCoR states and has been highly successful in enhancing research infrastructure in the EPSCoR jurisdictions (8, 9). Subsequently, many other federal agencies started EPSCoR programs, including the Department of Energy, Department of Defense, National Aeronautics and Space Administration, and Department of Agriculture.

Among the federal agencies that fund research, the National Institutes of Health (NIH), which has an annual budget exceeding $45 billion, is the largest. To promote geographic equity, NIH established its own program called the Institutional Development Award (IDeA) in 1993, which is similar to NSF’s EPSCoR program. The IDeA program’s goal was to increase the geographic distribution of NIH funding for biomedical and clinical research in approximately one-half of the states in the US, referred to as IDeA states, which were less competitive and secured less NIH funding than their counterparts. The EPSCoR and IDeA programs are similar in their overall objectives to enhance the research capacity in states that traditionally receive less NSF and NIH funding, respectively. While there is a significant overlap between EPSCoR- and IDeA-designated states, there are some states eligible for EPSCoR funding that are not eligible for IDeA funding and vice versa based on their success in securing NSF or NIH funding. Currently, the NIH IDeA program has allocated ~$430 million/year to support the IDeA states.

Although the EPSCoR and IDeA programs have been effective in providing additional resources to enhance research capacity in states that are eligible to receive such funding, whether such targeted funding has eliminated the geographic disparity in federal research funding is unclear. Recently, we demonstrated that non-EPSCoR states received significantly more research funding than the EPSCoR states from NSF (10). Interestingly, however, we found that for every dollar of federal research funding, the EPSCoR states performed better than the non-EPSCoR states in overall research productivity, as measured by the number of journal publications, books, conference papers, and citations (10).

There are many reasons why some states receive less NIH funding than others. One of these is that many IDeA states have small populations, thereby raising the question of whether federal funding for research should be based on a per-state or per-capita basis (11). Additionally, EPSCoR or IDeA states represent some of the poorest states in the US and receive only about 4% of the annual federal R&D investment but have nearly 20% of the U.S. population, thereby raising concerns about the disproportionate distribution of funds (12). To that end, in this study, we analyzed the population size of IDeA states vs. non-IDeA states and tried to determine if the NIH awards are proportional to the population size.

The National Academy of Sciences (NAS) has recommended that federal funding be provided to universities in all states so that students from all parts of the country have an opportunity to participate in high-quality research (11). NIH awards specific types of grants exclusively for training students and post-doctoral fellows (PDFs). Thus, one of the goals of this study was to analyze the number of PhD students and PDFs trained in IDeA and non-IDeA states and compare that to the awards made by NIH in individual and institutional training grants. Additionally, there is significant debate on whether the taxpayers who make the NIH funding feasible should expect spatial equality in NIH funding, especially because NIH funding is also associated with access to quality healthcare (13). In this study, we, therefore, tried to correlate the federal taxes paid by IDeA vs. non-IDeA states and the NIH funding received.

Our data demonstrated that NIH funding still remains concentrated in a few states. Thus, while the non-IDeA states together received almost 93.6% of NIH funds, the IDeA states received only 6.4%. Importantly, when we expressed the data on a *per capita* basis or based on the federal taxes contributed by the states, we found that IDeA states received disproportionately less NIH funds.

## Methods

### Data access and processing

The analysis data were extracted from various sources described below, each providing unique datasets associated with different states and years. These datasets were then aligned and merged based on the common identifier of state ID, ensuring consistency across the other sources. After merging, the consolidated data were further processed and organized using Excel to prepare it for subsequent analysis, which was done in R.

### IDeA and non-IDeA states

A list of IDeA and non-IDeA states as defined by the NIH was used to identify these groups of states, as shown in Table 1 (14), which included 23 IDeA states and 27 non-IDeA states. Although the NIH considers the Commonwealth of Puerto Rico to be one of the IDeA jurisdictions, we did not include this region in our study because of difficulties in capturing data.

**Table 1:** List of IDeA and non-IDeA states.

IDeA (23)	Non-IDeA (27)
Alaska	Alabama
Arkansas	Arizona
Delaware	California
Hawaii	Colorado
Idaho	Connecticut
Kansas	Florida
Kentucky	Georgia
Louisiana	Illinois
Maine	Indiana
Mississippi	Iowa
Montana	Maryland
Nebraska	Massachusetts
Nevada	Michigan
New Hampshire	Minnesota
New Mexico	Missouri
North Dakota	New Jersey
Oklahoma	New York
Rhode Island	North Carolina
South Carolina	Ohio
South Dakota	Oregon
Vermont	Pennsylvania
West Virginia	Tennessee
Wyoming	Texas
	Utah
	Virginia
	Washington
	Wisconsin

### NIH funding and categories

NIH’s Research Portfolio Online Reporting Tools (RePORT), available at https://report.nih.gov/ was used to extract data on the total NIH funding available by fiscal year from all institutes/centers, for all funding mechanisms, all congressional districts, and all organization types. Using the available tabs in RePORT, we selected to view this information by location https://report.nih.gov/award/index.cfm?ot=&fy=1992&state=USS,AL,AK,AZ,AR,CA,CO,CT,DE,DC,FL,GA,HI,ID,IL,IN,IA,KS,KY,LA,ME,MD,MA,MI,MN,MS,MO,MT,NE,NV,NH,NJ,NM,NY,NC,ND,OH,OK,OR,PA,RI,SC,SD,TN,TX,UT,VT,VA,WA,WV,WI,WY&ic=&fm=&orgid=&distr=&rfa=&om=n&pid=&view=statedetail#tab1.

To obtain individual category of grants such as non-SBIR/STTR, SBIR/STTR, individual training grants, institutional training grants, we used the same approach as described above except that we selected under “Funding Mechanism,” the specific categories of NIH grants listed above.

### Data on PhDs and PDFs trained

For PhD degrees awarded in IDeA vs. non-IDeA states, we used NSF’s Survey of Earned Doctorates (SED), available at https://ncses.nsf.gov/pubs/nsf23300/data-tables. We used the title “Doctorate institutions, locations, and countries of origins of research doctorate recipients,” and then Table 7–6 “State or location of doctorate institution ranked by the total number of research doctorate recipients, by field of doctorate and sex” and then Table B-1 https://ncses.nsf.gov/pubs/nsf23300/technical-notes#technical-tables, which shows the crosswalk of the tables to previous years. For analysis of the number of PDFs trained, the data were downloaded from the National Center for Science and Engineering Statistics (NCSES): https://ncsesdata.nsf.gov/builder/gss.

#### Data on population of IDeA *vs* non-IDeA states

The data on population in IDeA vs. Non-IDeA was obtained from https://www2.census.gov/programs-surveys/popest/tables/2020-2023/state/totals/NST-EST2023-POP.xlsx

#### Data on federal taxes paid by IDeA *vs* non-IDeA states

The data on gross federal tax collections from IDEA vs. non-IDEA states was collected from the Internal Revenue Service document https://www.irs.gov/pub/irs-prior/p55b--2022.pdf

### Statistical analysis

The data, including total NIH awards, non-SBIR/STTR grants, SBIR/STTR grants, individual training grants, institutional training grants, number of PhDs and PDFs trained, population, and total federal tax revenues from IDeA vs. non-IDeA states were compared, and expressed in two formats in Figures. First, we calculated and depicted the mean values for IDeA and non-IDeA states. Second, the data were expressed as Mean+/−SD for each of the two groups, and these values were compared for statistical significance using a *t*-test. The *p* values were depicted in each figure and *p* < 0.05 was considered statistically significant.

Additionally, we examined the association between total NIH awards, non-SBIR/STTR, SBIR/STTR, individual training grants, institutional training grants, and IDeA/non-IDeA using the univariate regression (Supplementary Table 1). In this analysis, we did not include PhDs and PDFs trained, state population, and federal taxes collected as confounders because these are uncorrelated with NIH funding. *p* < 0.05 was considered to be statistically significant.

## Results

### Comparing total NIH funds received by IDeA and non-IDeA states

**Figure 1:**
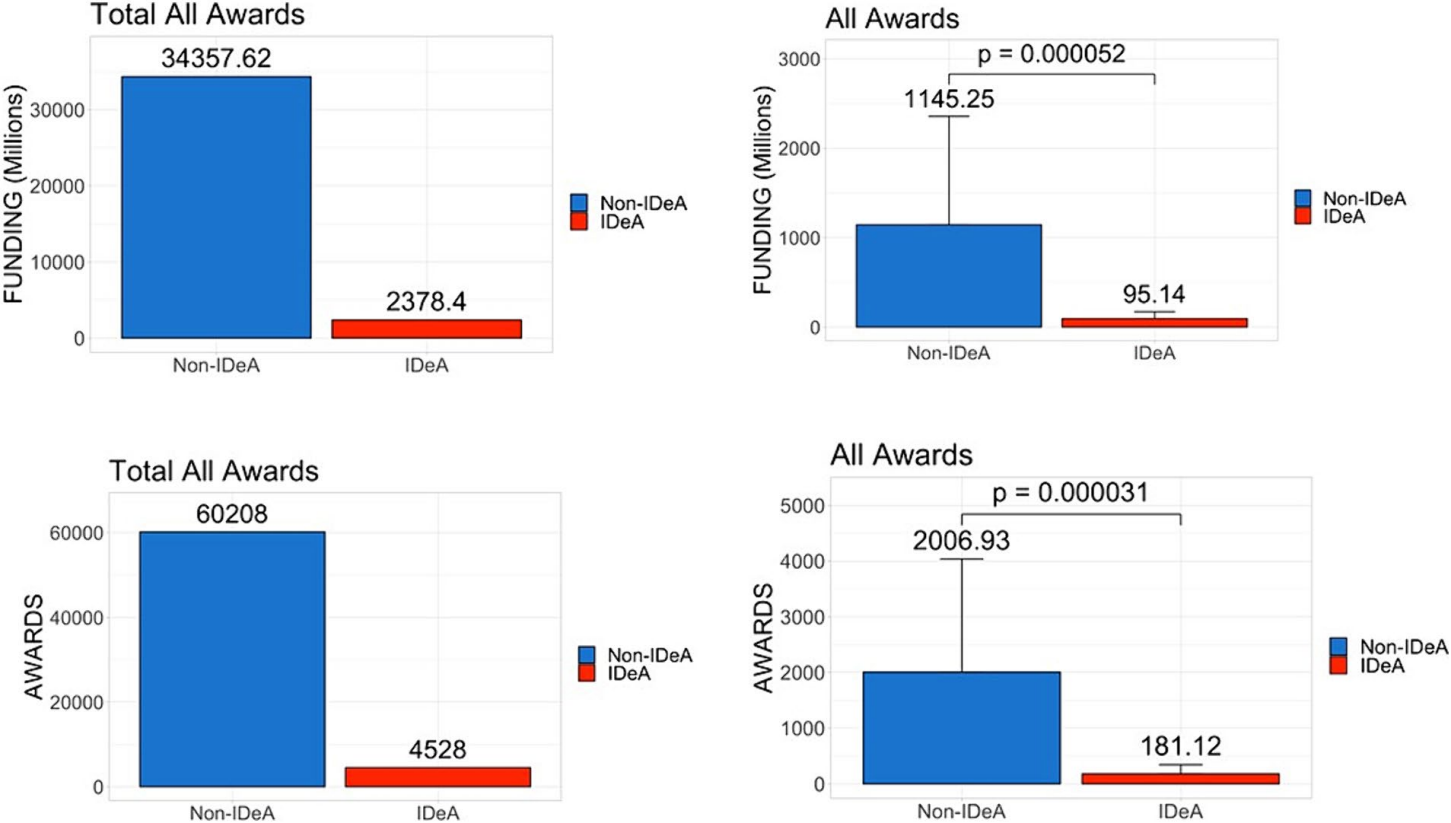
Distribution of all NIH grant awards across IDeA vs. non-IDeA states. The upper panels depict the dollar amounts of all NIH funds received, and the lower panels show the number of NIH grant awards to IDeA vs. non-IDeA states. The right panels show Mean+/−SD with the Mean values depicted on top of the vertical bars.

To compare the NIH funds received by IDeA vs. non-IDeA states, we analyzed the NIH Report published data for FY 2022 on all NIH awards, which included research project grants (including SBI/STTR and non-SBIR/STTR), Research Center grants, Individual and Institutional Training grants, R&D contracts and construction grants, and other grants as defined by NIH. We noted that in 2022, the non-IDeA states received a total of $34.35 billion (93.6%) compared to the IDeA states that received only $2.37 billion (6.4%) (Figure 1). The per-state mean value of these total NIH grants for non-IDeA states was $1.14 billion when compared to the IDeA states that received $0.09 billion per state, and these differences were statistically significant (Figure 1). The lower panels in Figure 1 show that the total number of NIH awards received by IDeA states (60,208)(93%)was much less than that received by non-IDeA states (4,528) (7%), and the difference in the mean number of awards per state in IDeA vs. non-IDeA groups was statistically significant. Together, these data demonstrated that the non-IDeA states received substantially more funds and a greater number of grants (93–94%) than the IDeA states received (6–7%).

### Comparing different categories of NIH funds received by IDeA and non-IDeA states

**Figure 2:**
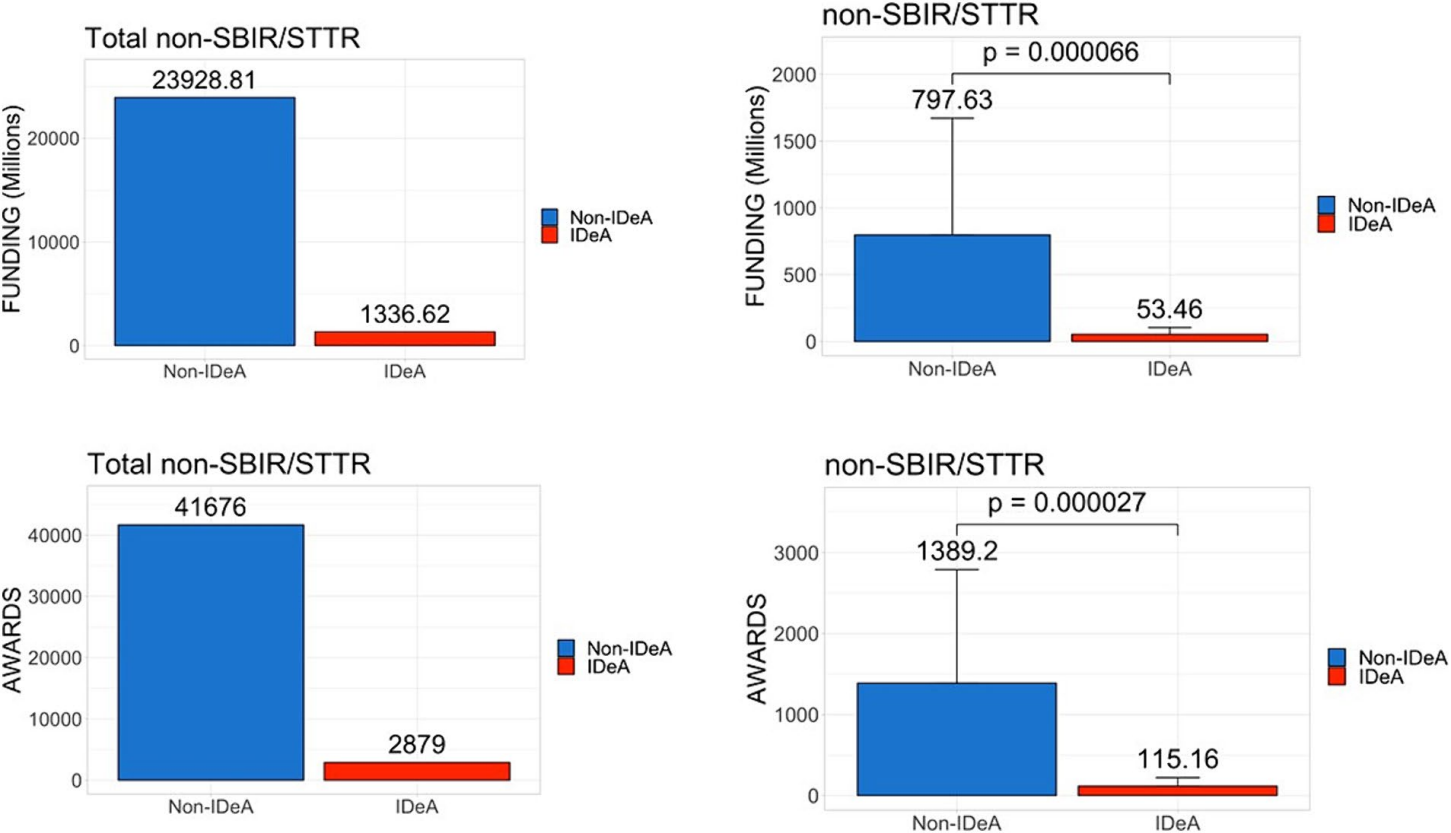
Distribution of non-SBIR/STTR awards across IDeA vs. non-IDeA states. The upper panels show the dollar amounts of non-SBIR/STTR funds received, and the lower panels show the number of non-SBIR/STTR grants awarded made to IDeA vs. non-IDeA states. The right panels show Mean+/−SD with the Mean values depicted on top of the vertical bars.

**Figure 3:**
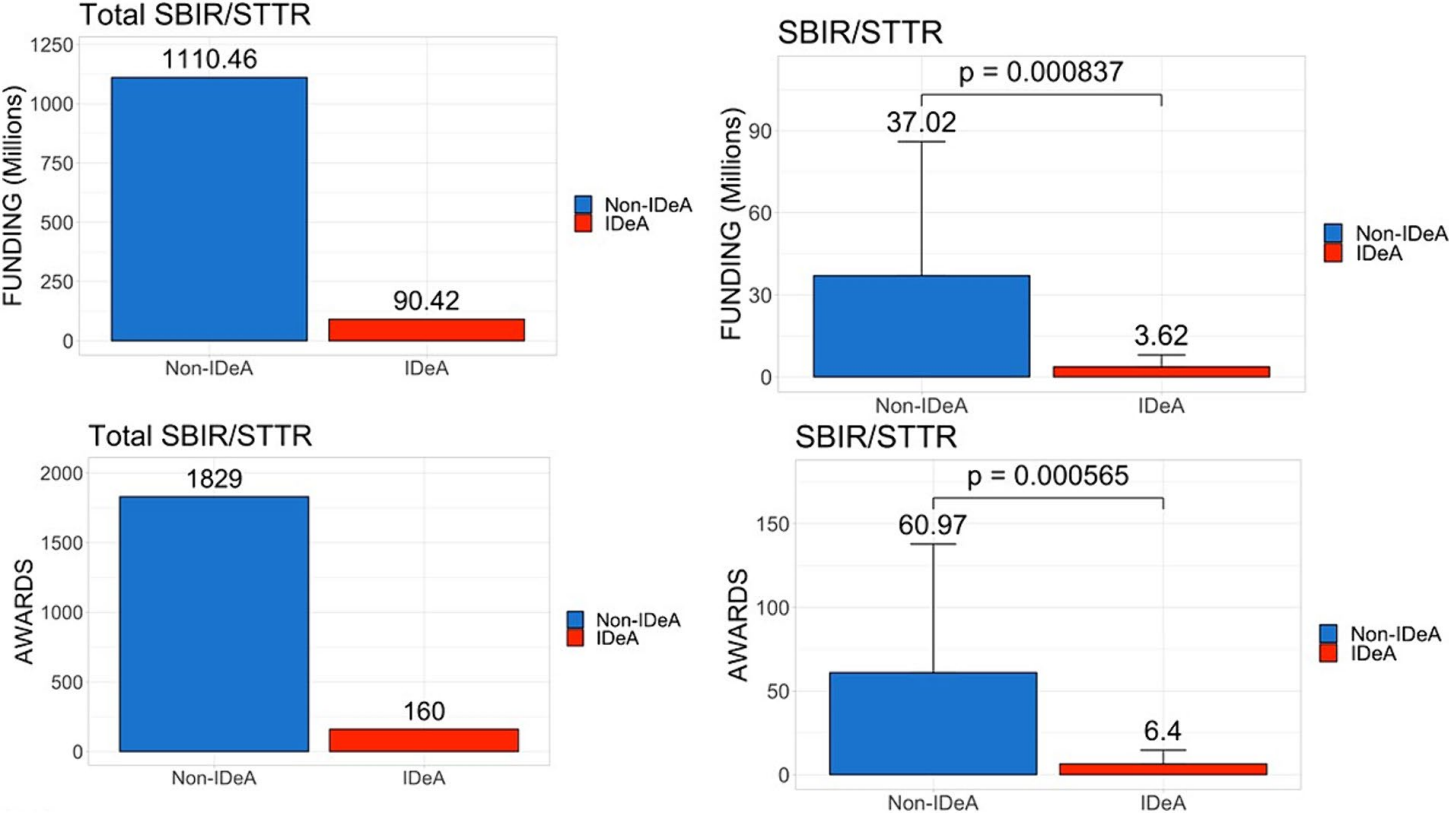
Distribution of SBIR/STTR awards across IDeA vs. non-IDeA states. The upper panels show the dollar amounts of SBIR/STTR funds received, and the lower panels show the number of SBIR/STTR grants awarded to IDeA vs. non-IDeA states. The right panels show Mean+/−SD with the Mean values depicted on top of the vertical bars.

To test if there are any differences in the awards in different categories of NIH grants received by various states, we next analyzed the data for both non-SBIR/STTR and SBIR/STTR funding mechanisms for FY 2022. The data in Figure 2 demonstrated that the non-IDeA states received $23.92 billion (94.7%) in non-SBIR/STTR funding compared to the IDeA states that received $1.33 billion (5.3%). In addition, the non-IDeA states received a larger number of non-SBIR/STTR grants than the IDeA states. Both the dollar amounts and the number of grants received in this category were statistically different in IDeA vs. non-IDeA states (Figure 2, lower panels). When we analyzed the data for SBIR/STTR grants (Figure 3), we found a similar pattern: non-IDeA states received significantly more SBIR/STTR grants ($1,110 million, 92.5%) than IDeA states ($90 million, 7.5%).

**Figure 4:**
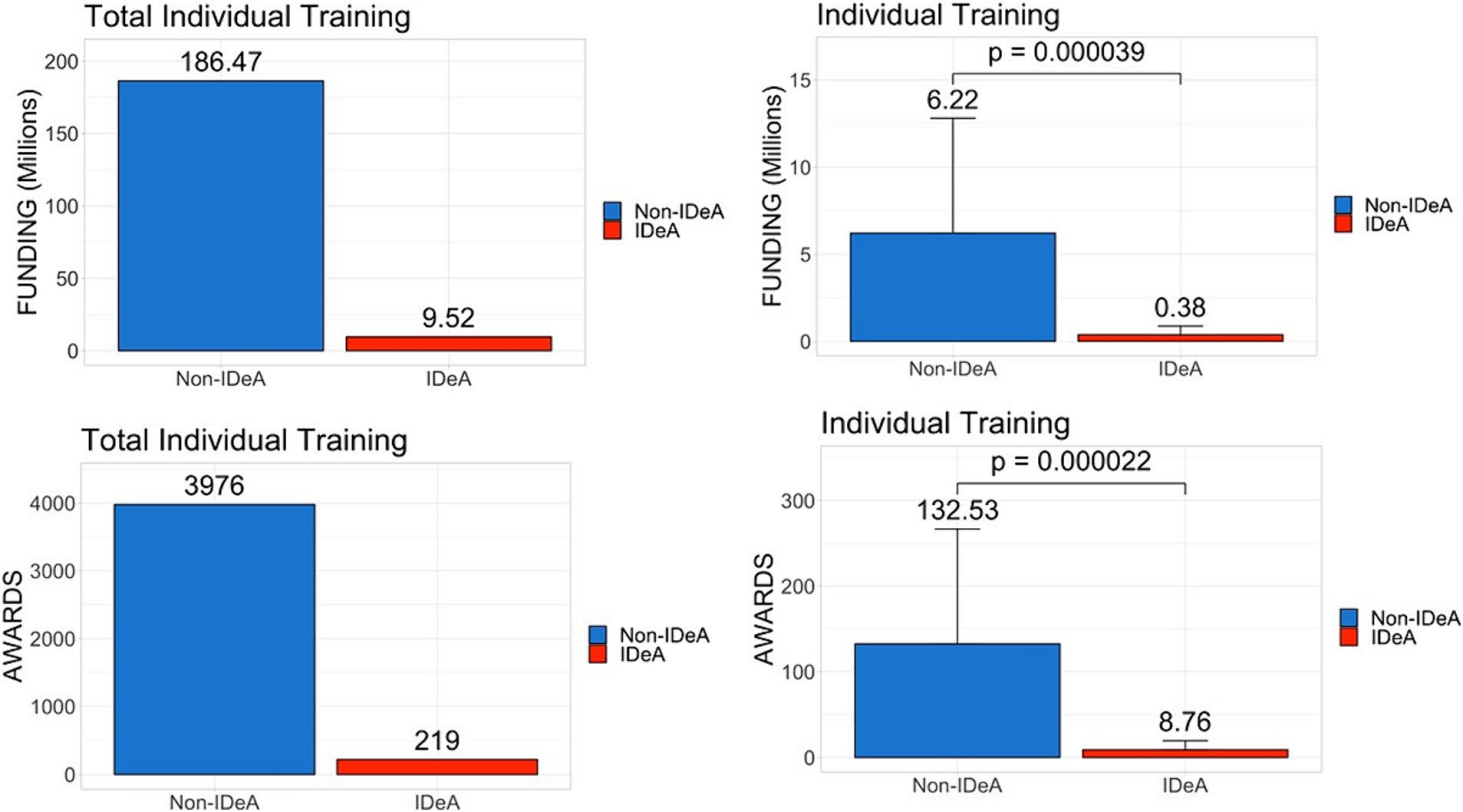
Distribution of individual training grants awarded across IDeA vs. non-IDeA states. The upper panels show the dollar amounts of individual training grants awarded, and the lower panels show the number of individual training grants awarded to IDeA vs. non-IDeA states. The right panels show Mean+/−SD with the Mean values depicted on top of the vertical bars.

**Figure 5:**
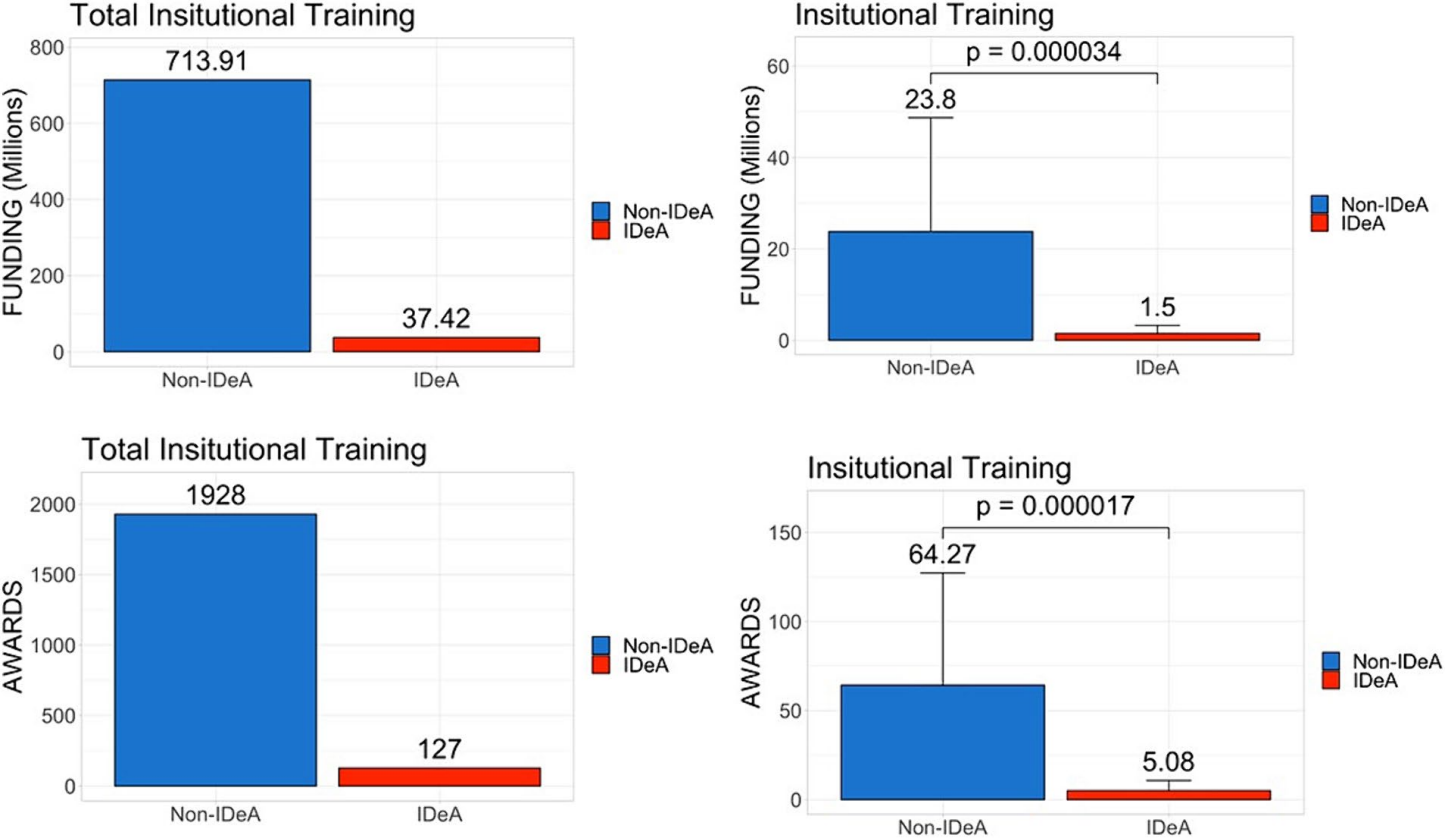
Distribution of institutional training grants awarded across IDeA vs. non-IDeA states. The upper panels show the dollar amounts of institutional training grants awarded, and the lower panels show the number of institutional training grants awarded to IDeA vs. non-IDeA states. The right panels show Mean+/−SD with the Mean values depicted on top of the vertical bars.

Next, we analyzed data for individual training grants that are used primarily to support trainees at the graduate and postdoctoral levels (Figure 4), and institutional training grants (Figure 5) that are awarded to institutions to provide financial support and mentoring to undergraduate, graduate, and postdoctoral scholars. These data demonstrated that there was a significant disparity between IDeA and non-IDeA states, with the latter receiving a larger number of these grants and much higher levels of funding than the former group (Figures 4, 5, statistical significance shown in right panels). Additionally, when the total number of awards in each category was calculated, we found that non-IDeA states performed significantly better than IDeA states.

### Number of PhDs and post-doctoral fellows (PDFs) trained in IDeA vs. non-IDeA states

**Figure 6:**
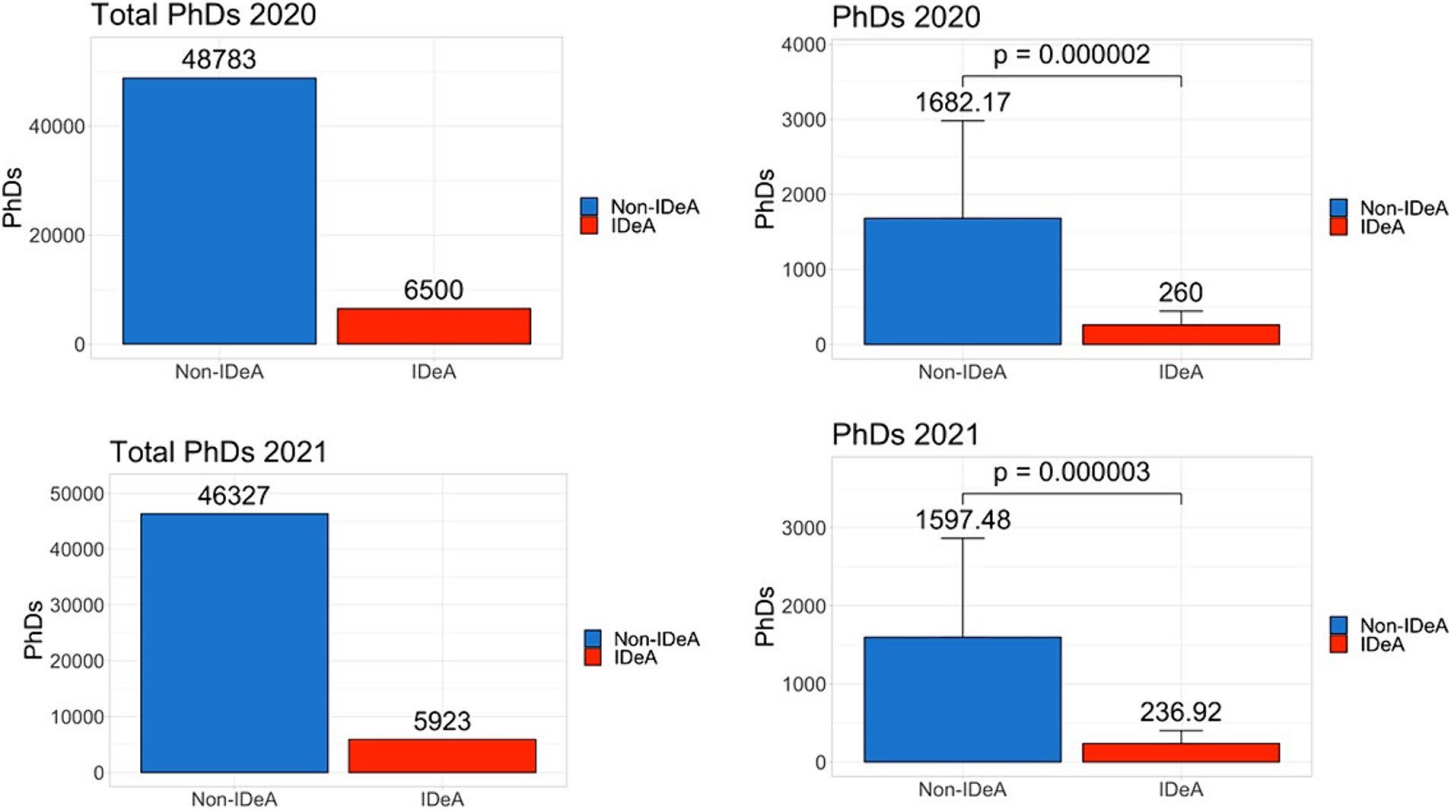
Number of PhD degrees awarded in non-IDeA and IDeA states. The upper panels show PhD degrees awarded in 2020 and the lower panels, in 2021. The right panels show Mean+/−SD with the Mean values depicted on top of the vertical bars.

**Figure 7:**
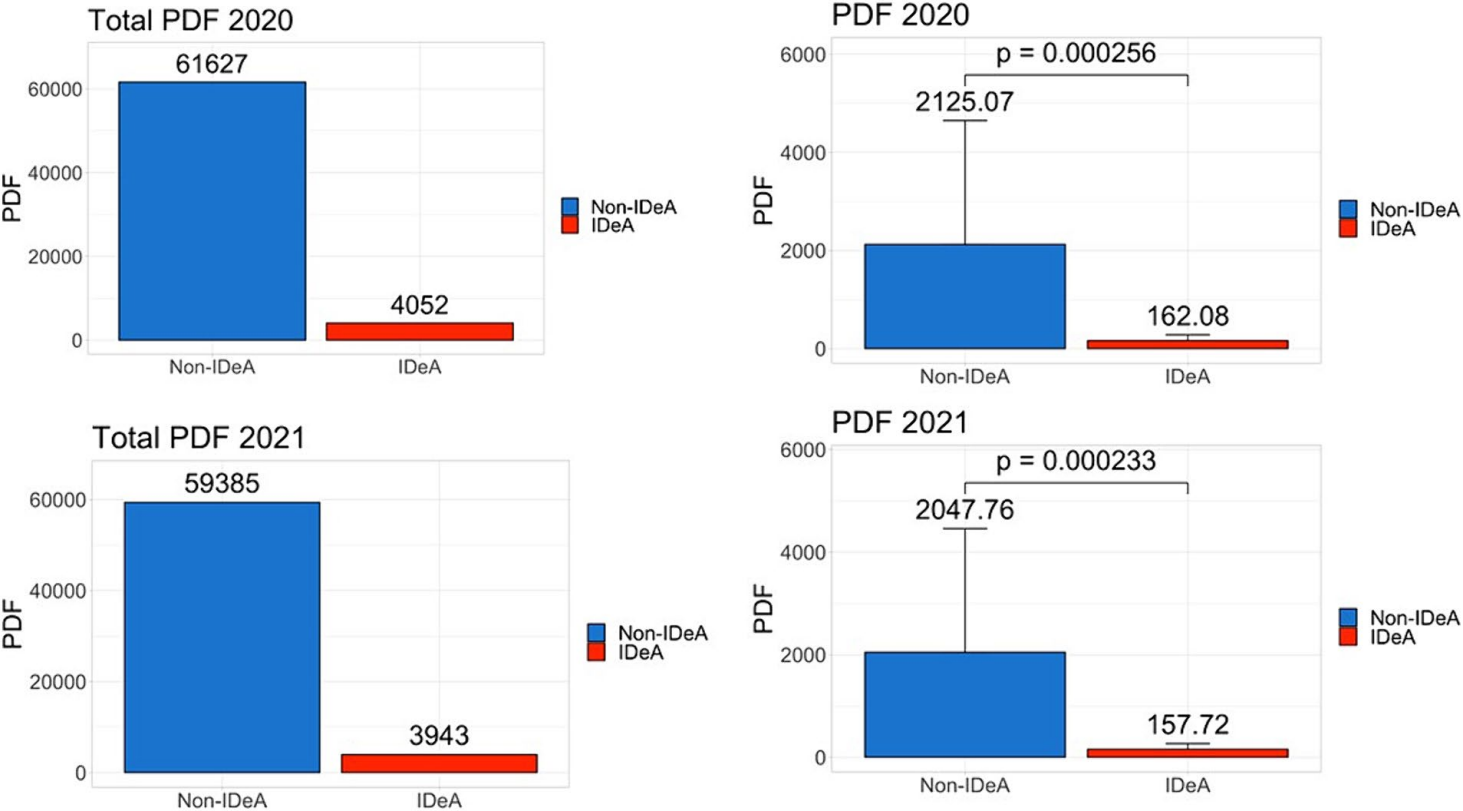
Number of Post-doctoral Fellows (PDFs) employed in non-IDeA and IDeA states. The upper panels show PDFs employed in 2020 and the lower panels, in 2021. The right panels show Mean+/−SD with the Mean values depicted on top of the vertical bars.

Because NIH funds PhD and Post-doctoral trainees, we next determined the number of PhDs and PDFs trained in IDeA vs. non-IDeA states. The data demonstrated that in 2020 and 2021, non-IDeA states trained more PhDs and PDFs than IDeA states, and these differences were statistically significant (Figures 6, 7). For example, in 2020, non-IDeA states trained 48,783 (88.2%) PhDs, whereas IDeA states trained 6,500 (11.8%) PhDs (Figure 6). Similarly, in 2020, non-IDeA states trained 61,627 (93.8%) PDFs, whereas IDeA states trained 4,052 (6.2%) PDFs (Figure 7). When the data were expressed as the number of PhDs or PDFs trained per state, it was evident that the non-IDeA states trained more PhDs or PDFs than the IDeA states, which was statistically significant. It is also worth noting that the number of PhDs and PDFs trained in 2020 and 2021 was similar.

### Population in IDeA and non-IDeA states

**Figure 8:**
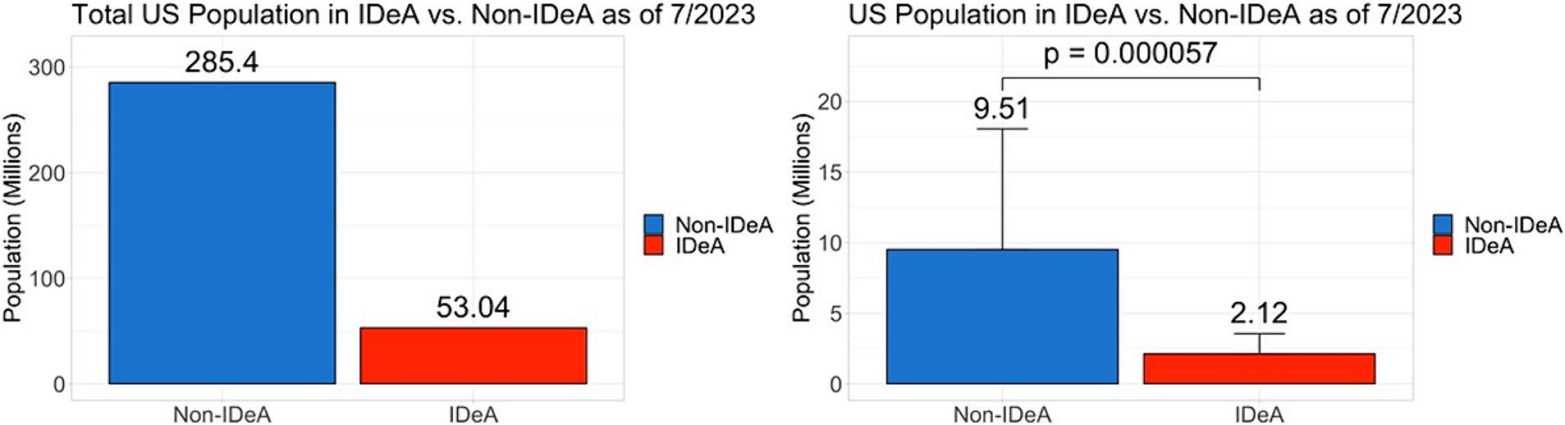
Population in non-IDeA and IDeA states. The right panels show Mean+/−SD with the Mean values depicted on top of the vertical bars.

One reason why non-IDeA states receive significantly higher levels of funding than IDeA states could be that they are more densely populated. To that end, we analyzed the population data as of 2023 and found that the non-IDeA states had a total of 285.4 (84.3%) million people compared to the IDeA states, which had only 53.04 (15.7%) million (Figure 8, statistical significance shown in left panel). On average, non-IDeA states had 9.51 million people per state, whereas IDeA states had 2.12 million people per state.

### Federal taxes paid by non-IDeA states versus IDeA states

**Figure 9:**
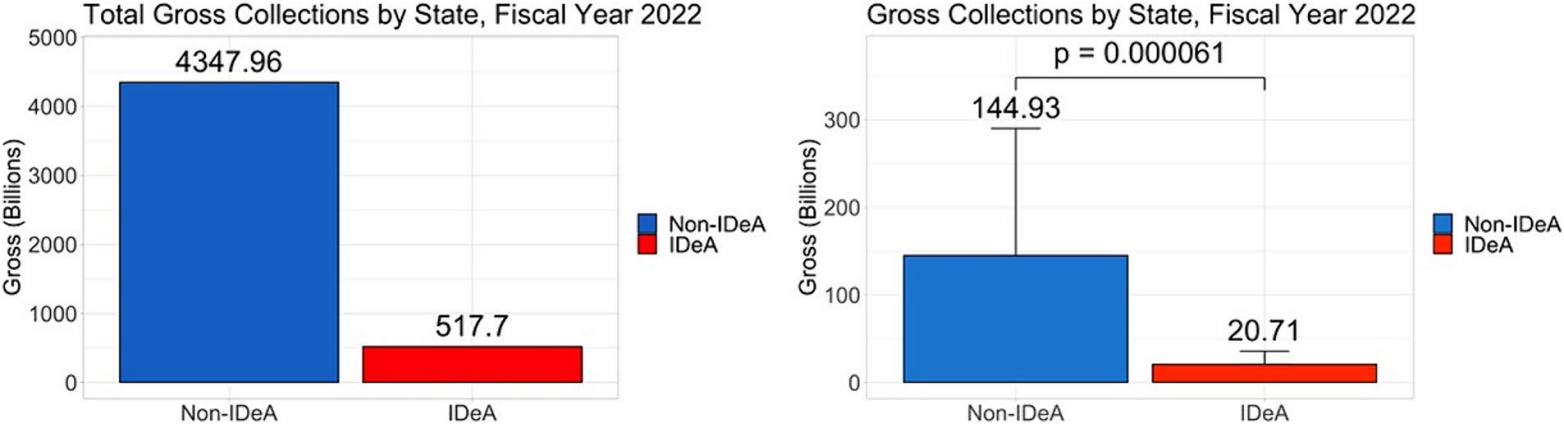
Total federal taxes paid by non-IDeA vs. IDeA states. The right panels show Mean+/−SD.

Another possibility for the geographic disparity in funding could result from the total federal taxes collected from these states. To that end, we analyzed total gross federal taxes collected from non-IDeA vs. IDeA states. We found that non-IDeA states contributed $4,347 billion (89.4%) in federal taxes compared with IDeA states which contributed $517 billion (10.6%) (Figure 9), which was statistically significant (Figure 9, right panel).

### Total NIH funding to non-IDeA states versus IDeA states in years 1992, 2012, and 2022

**Figure 10:**
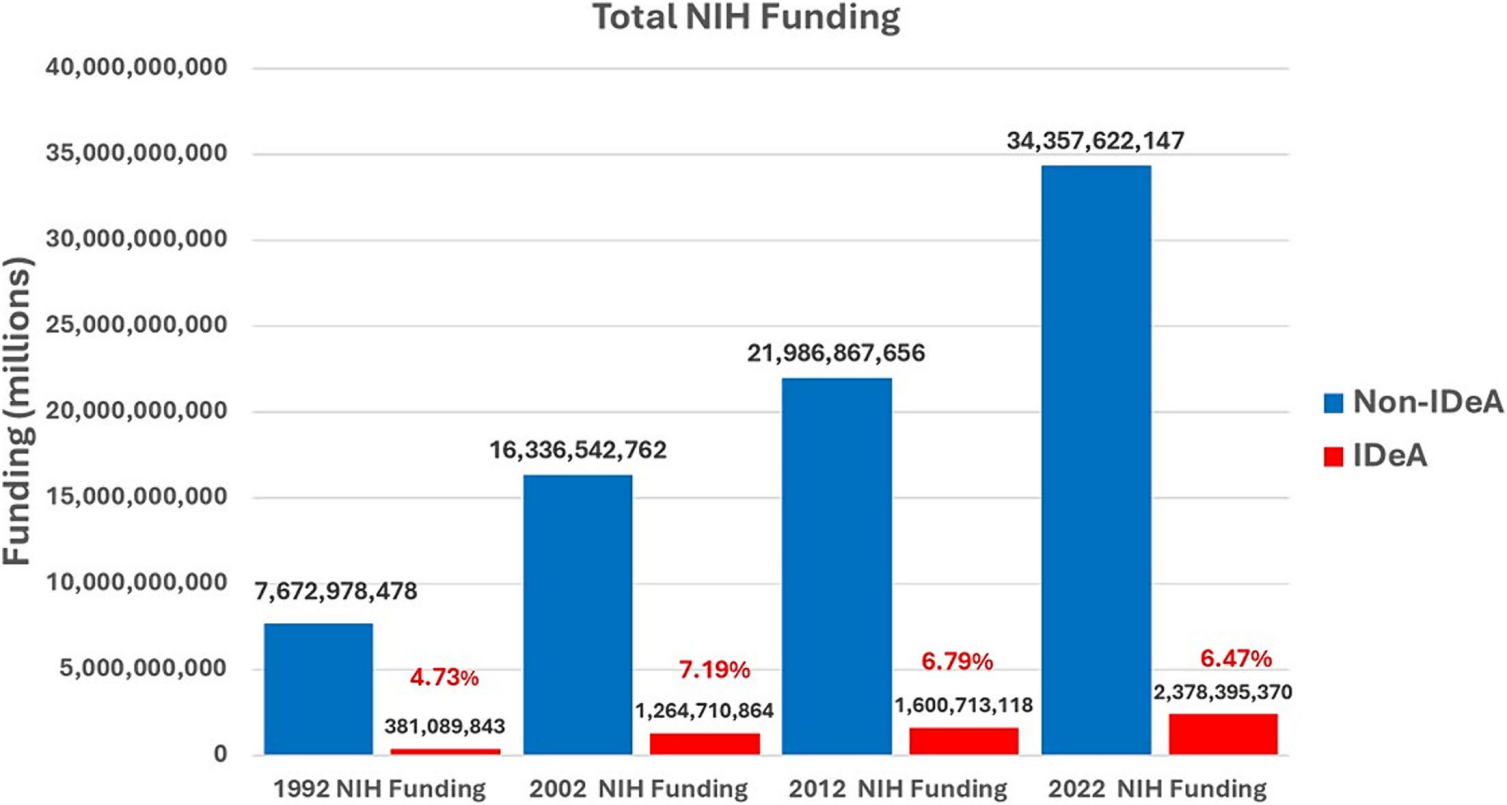
Total NIH funding received by non-IDeA vs. IDeA states in 1992, 2002, 2012, and 2022. The percentage of funding received by IDeA states has been depicted in red font.

The IDeA program was initiated in 1993. Therefore, we analyzed the data for FY 1992 to measure the level of total awards to non-IDeA vs. IDeA states before the IDeA programs were started. Additionally, we analyzed the data for 2012 and 2022 to study the trend in NIH funding across 4 decades. The data showed that in 1992, the non-IDeA states received $7.67 billion (95.3%) while IDeA states received $0.38 billion (4.73%) (Figure 10). Thus, the IDeA states received ~4.7% of total NIH funding compared to 2022 (Figure 1), when they received ~6.4%. From 1992 to 2022, the NIH funding grew substantially; however, the proportion of funds received by IDeA vs. non-IDeA states remained relatively the same (Figure 10). From 1992 to 2002, the IDeA state saw a slight increase in NIH funding from 4.73 to 7.19% of the total budget. However, from 2002 to 2022, there was, in fact, a slight decrease in the proportion of NIH funding received by the IDeA states from 7.19% in 2002 to 6.79% in 2012 and to 6.47% in 2022.

### Examining the data on NIH funding to IDeA vs. non-IDeA states using the univariate regression model

While we compared all data between IDeA vs. non-IDeA using the t-test as described above, we also performed univariate regression analysis (Supplementary Table 1). These findings were consistent with the t-test analysis. For example, all NIH funding was $1050.12 million [95% confidence interval (CI): (-2624.26, -1027.37)], significantly lower compared to the non-IDeA states (*p* = 0.000070). Also, NIH awards were 1050.12 (95% CI: (−2624.26, −1027.37), significantly lower than non-IDeA states (*p* = 0.000040). These findings are consistent with the *t*-test (Figure 1). Similar observations were made using data from non-SBIR/STTR, SBIR/STTR, individual training grants, and institutional training grants (Supplementary Table 1).

## Discussion

The present study demonstrates a significant disparity in NIH funding received by non-IDeA states versus IDeA states in the US. The non-IDeA states, which constitute ~54% of the 50 states in the US, received 93.6% of NIH funding, whereas the IDeA states received only 6.4%. This was seen not only with total NIH grants but also among different categories of NIH grants, such as non-SBIR/STTR SBIR/STTR, institutional training grants, and individual training grants.

One can speculate many reasons why non-IDeA states receive higher levels of research funding: (1) the economy of the state, (2) the population size of the state, (3) state investments in research infrastructure, (4) the number of Carnegie R1 universities, (5) the federal tax revenues generated from the state, (6) presence of academic health science centers, etc. Interestingly, when we studied the population in IDeA vs. non-IDeA states, we found that the IDeA states had 15.7% of the U.S. population compared to the non-IDeA states, which had 84.3%. In contrast, the IDeA states received only 6.4% of total NIH funding, whereas non-IDeA states received 93.6% of the funding. Thus, on a *per capita* basis, non-IDeA states received $120 from NIH, whereas IDeA states received $45 per person. Thus, the increased funding from NIH to non-IDeA states did not correlate with the states’ population. The fact that *per capita*, the IDeA states receive significantly less NIH funding also translates into fewer healthcare benefits because NIH funding supports not only research and training but also clinical trials, early diagnosis of diseases, and access to advanced care, such as that seen at National Cancer Institute (NCI)-designated Cancer Centers. In fact, several IDeA states do not have such NIH-funded Cancer Centers (15).

The geographic disparity in NIH funding may also result from differences in the federal tax base of the IDeA and non-IDeA states. To that end, we analyzed the total taxes paid by the IDeA vs. non-IDeA states and found that non-IDeA states together contributed toward federal taxes $4,347 billion which constituted 89.4% when compared to the IDeA states which contributed $517 billion (10.6%). Thus, for every million dollars contributed by the non-IDeA states toward federal taxes, they received $7,903 in NIH funds, whereas for every million dollars contributed by the IDeA States in taxes, they received only $4,599. Thus, on a per-dollar basis of federal taxes paid, IDeA states received significantly less NIH funding than non-IDeA states.

The National Academy of Sciences has recommended that federal funding be provided to students from all parts of the country so that they get an opportunity to pursue high-quality research (11). To that end, we calculated the number of individual and institutional training grants awarded and compared that to the number of PhDs and PDFs trained. In non-IDeA states, in 2021, 46,327 PhDs were awarded, and they received 1928 institutional training grant awards. In contrast, in IDeA states, there were 5,923 PhDs and 127 institutional training grant awards. Thus, in non-IDeA states, there was one institutional training grant for every 24 PhD trainees, while in IDeA states, there was one institutional training grant for every 46 students. This clearly demonstrated that PhD trainees enrolled in IDeA states are at a disadvantage in receiving NIH-funded predoctoral traineeships. This also prevents the IDeA states from attracting the best PhD trainees who may prefer to join a PhD program that offers an NIH Institutional training program. This disparity was also seen with individual training grants that were awarded in greater numbers to the non-IDeA states compared with the IDeA states, thereby providing better opportunities for doctoral and post-doctoral trainees in non-IDeA states. To check if the NIH funding correlates with the numbers of Carnegie-classified R1 universities with Very High Research Activity, we investigated the number of such institutions in IDeA vs. non-IDeA states. We found that in the US, there were a total of 146 R1 universities, of which 119 were located in non-IDeA states, and 27 were found in IDeA states (16). Thus, in theory, each R1 university in a non-IDeA state received $288 million, while each R1 institution in an IDeA state received $88 M. Thus, the NIH funding did not correlate with the number of R1 universities in IDeA vs. non-IDeA states.

Concerning SBIR/STTR funding, these programs provide funding to small businesses and to academic institutions to pursue research and technological innovation to help grow regional and national economies. Thus, receiving only 7.5% of the SBIR/STTR funding by the IDeA states clearly makes them less competitive in helping to grow the regional economy. The NIH has estimated that every $100 million in NIH funding, creates 76 patents, and furthermore, such patents create an additional $598 million in further research and development (17). NIH-funded patents are believed to have 20% more economic value than other U.S. patents. Thus, receiving low SBIR/STTR funding negatively impacts the IDeA states’ ability to grow their regional economies. This also supports our previous studies in which we found that for every dollar invested by federal agencies, EPSCoR states performed better in all research metrics except the patent category, where non-IDeA states performed better than IDeA states (10).

The NIH estimates that every dollar of NIH funding generates approximately $2.64 of economic activity (17). Thus, in 2022, NIH funding generated an estimated $96.9 billion in economic activity. Considering this, the non-IDeA states had an economic activity of $90.6 Billion when compared with the IDeA states, which had an economic activity of $6.3 billion.

While special programs such as EPSCoR started by most federal agencies, including the NSF, and IDeA started by the NIH over the past several decades have certainly helped EPSCoR/IDeA states grow their research infrastructure and capacity, the funding allocated to these programs is a small fraction of the total budget. For example, the NSF EPSCoR program budget for fiscal year 2023 was $245 million, whereas the entire NSF budget for that year was $9.877 billion. Thus, NSF allocated 2.48% of its budget to support EPSCoR programs across eligible jurisdictions. Similarly, NIH allocated $425 million for the IDeA program in 2023, when its total budget was $47.5 billion, constituting only 0.89% of the total NIH budget. Because of the disproportionate levels of NSF funding to the EPSCoR states, the Congress, in the CHIPS and Science Act of 2022, directed NSF to set aside 20% of its R&D budget to support research at EPSCoR states (18).

One of the limitations of the current study is that our data did not capture multiple-principal investigator (PI) grants in which one of the contact PIs was from an IDeA state, while the other PI was from a non-IDeA state or vice versa, and assign precise dollar amounts to these states. Similarly, the NIH Reporter did not allow us to gather data when a collaborator (Co-investigator) was from a non-IDeA state or vice versa.

In summary, the gap in NIH funding between IDeA and non-IDeA states persists. This continues to have damaging effects on IDeA states in terms of training future generations of scientists and innovators, the regional economy, research and healthcare jobs, and access to innovative treatments for patients. IDeA states are primarily rural and medically underserved regions (19) and thus, lower levels of NIH funding negatively impact the health outcomes for their populations. This also makes it challenging for IDeA states to attract and retain their top-tier researchers, graduate students, and PDFs.

Actionable recommendations: the current commitment from NIH to the IDeA programs is less than 1% of the NIH budget. Thus, its impact on healthcare and biomedical research in IDeA states is limited in terms of direct and immediate effects. NIH should consider enhancing the funding for IDeA states through sustained increases in IDeA programs or other measures similar to those enacted by Congress to increase NSF funding through the CHIPS and Science Act.
