# Supplementary figures and images for: Geographic inequality in funding by National Institutes of Health negatively impacts almost one-half of the states in the United States
Source: Front Public Health. 2024 Sep 25;12:1452494. doi: 10.3389/fpubh.2024.1452494 (PMC11461335; doi:10.3389/fpubh.2024.1452494)

Supplemental Table 1: Examination of the association using the univariate regression model.


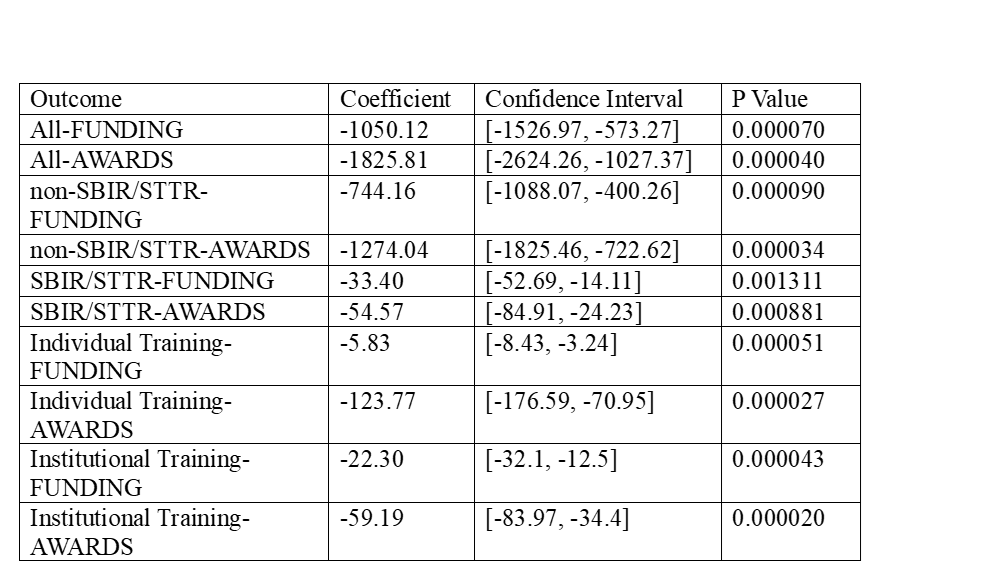

Supplement: Supplementary file 1 [file Table_1.DOCX]
